# Adhesive capsulitis after COVID-19 vaccine injection: a peculiar case treated with combined bursa distention and glenohumeral capsular hydrodilatation

**DOI:** 10.1007/s40477-022-00739-3

**Published:** 2023-01-03

**Authors:** Alessandro Biglia, Valentina Morandi, Giovanni Zanframundo, Danilo Donati, Francesco Maggiore, Fabio Vita, Luigi Sammarchi, Chiara Pagani, Lorenzo Cavagna, Stefano Galletti, Carlomaurizio Montecucco

**Affiliations:** 1 Department of Internal Medicine and Therapeutics, Università di Pavia, Pavia, Italy; 2 Division of Rheumatology, Fondazione IRCCS Policlinico San Matteo, V. le Golgi 19, 27100 Pavia, Italy; 3 Physical Medicine and Rehabilitation Unit, IRCCS-Istituto Ortopedico Rizzoli, Bologna, BO Italy; 4 Department of Orthopedic and Traumatological Surgery, Istituto Ortopedico Rizzoli, Bologna, Italy; 5 Department of Diagnostic and Interventional Radiology and Neuroradiology, IRCCS San Matteo University Hospital Foundation, Pavia, Italy; 6 Musculoskeletal Ultrasound School, Italian Society for Ultrasound in Medicine and Biology, Bologna, Italy

**Keywords:** Adhesive capsulitis, SIRVA, Ultrasound, US-guided capsule distension

## Abstract

Frozen shoulder is a common and self-limiting condition affecting the soft tissues of the shoulders, characterized by severe pain, impaired range of motion (ROM) and limitation of daily activities. Its prevalence is 5% and it occurs most commonly in the fifth and sixth decades of life; women are more affected [DePalma in Clin Orthop Relat Res 466:552–560, 2008]. It can be idiopathic or associated with other conditions such as metabolic disorders, diabetes, thyroid diseases, prolonged immobilization, trauma [DePalma in Clin Orthop Relat Res 466:552–560, 2008], or complications after vaccine administration known as SIRVA (Shoulder injury related to vaccine administration). SIRVA is not caused by the vaccine itself but by inappropriate vaccination techniques [Martín Arias et al. in Vaccine 35:4870–4876, 2017]. The natural history of the frozen shoulder is a progression through three stages based on clinical and arthroscopic presentations: freezing, frozen and thawing [DePalma in Clin Orthop Relat Res 466:552–560, 2008; Do et al. in Orthop J Sport Med 9:232596712110036, 2021]. The onset is characterized by disabling pain, that worsens at night; it is induced by inflammation and hypervascularity and lasts from 10 to 36 weeks [Do et al. in Orthop J Sport Med 9:232596712110036, 2021]. The second stage is predominated by stiffness and severe reduction of ROM. This phase typically lasts from 9 to 12 months [Do et al. in Orthop J Sport Med 9:232596712110036, 2021]. Eventually, a recovery phase occurs, with a gradual recovery of the ROM that can last between 12 and 42 months. Ultrasound is an emerging diagnostic tool that contributes to differential diagnosis and treatment [Zappia et al. in Insights Imaging 7:365–371, 2016; Ricci et al. in J Ultrasound Med 39:633–635, 2020]: signs of adhesive capsulitis consist of thickening of the inferior recess of the glenohumeral joint capsule, thickening of the coracohumeral ligament and soft tissue structures in the rotator cuff interval, with hypervascularity. An unspecific sign is increased fluid in the tendon sheath of the long head of the biceps [Martín Arias et al. in Vaccine 35:4870–4876, 2017; Tandon et al. in J Ultrasound 20:227–236, 2017].

## Case report

We present a case regarding a 50-year-old female patient, non-diabetic woman with no history of thyroid or autoimmune disease, who developed adhesive capsulitis and subacromial-subdeltoid (SASD) fibro-adhesive bursitis 48 h after the second dose of anti-Sars-CoV-2 vaccine injection (Comirnaty) into the left shoulder [2, 7]

Symptoms persisted and worsened over the next four weeks, so the patient performed blood tests such as complete cell blood count (CBC), c-reactive protein (CRP) and erythrocyte sedimentation rate (ESR), which resulted to be normal. Clinically, the abduction and extra rotation movements were severely impaired so that the patient couldn’t raise her arm upon her shoulder. The ultrasound (US) examination was performed, showing no signs of lesions of the rotator’s cuff tendons. However, the rotator interval was thickened compared to the contralateral one (2.3 mm at right shoulder vs 3.3 mm at the left shoulder) (Panel 1A and B). A thin layer of fluid effusion in the most declivous part of the long head of the biceps was present and an inferior recess of the glenohumeral capsule thickening was present at the longitudinal scan of the axillary cavity (2.7 mm at right shoulder vs 6,4 mm at the left shoulder) (Panel 1C and D). Furthermore, there was the presence of SASD fibro-adhesive bursitis (Panel 2A). The diagnosis of adhesive capsulitis with associated adhesive bursitis was made and as first-line therapy, the patient assumed an oral nonsteroidal anti-inflammatory drug (Etoricoxib 90 mg daily) for ten days, without benefit. Therefore, the patient underwent two US-guided capsule hydrodistention with saline (8 mL), methylprednisolone 40 mg (1 mL), hyaluronic acid (2 mL) and lidocaine (2 mL) combined to SASD hydrodilatation with 4 mL of saline solution and 1 mL of lidocaine (Panel 2B), two weeks apart [8]. To break-up fibrotic adhesion the procedure was followed by 30 min of passive mobilization including intra-rotation, extra-rotation, adduction, abduction, flexion, extension and finally with Codman pendulum exercise. The patient continued to perform physiotherapy at home, twice a week, with the help of a physical therapist, between the first and second session and after the second one. After 4 weeks, the pain resolved and the ROM was almost completely recovered. A follow-up ultrasound was performed three months later and revealed a reduction of the capsule and rotator interval thickness and a resolution of the SADS bursitis [8, 9]. The third administration of COVID-19 vaccine (Comirnaty) performed in the contralateral shoulder did not create any more complications for the patient. Frozen shoulder is believed to be a self-limiting condition, even though the complete resolution does not occur in many patients.

**Panel 1:**
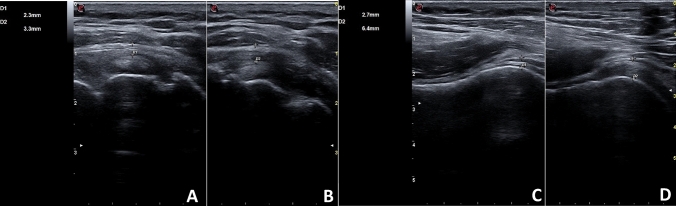
**A** Normal rotator cuff interval (right shoulder); **B** rotator cuff interval thickened (left shoulder); **C** normal inferior glenohumeral recess capsule (right axillary cavity US longitudinal scan); **D** thickened inferior glenohumeral recess capsule (left axillary cavity US longitudinal scan)

**Panel 2:**
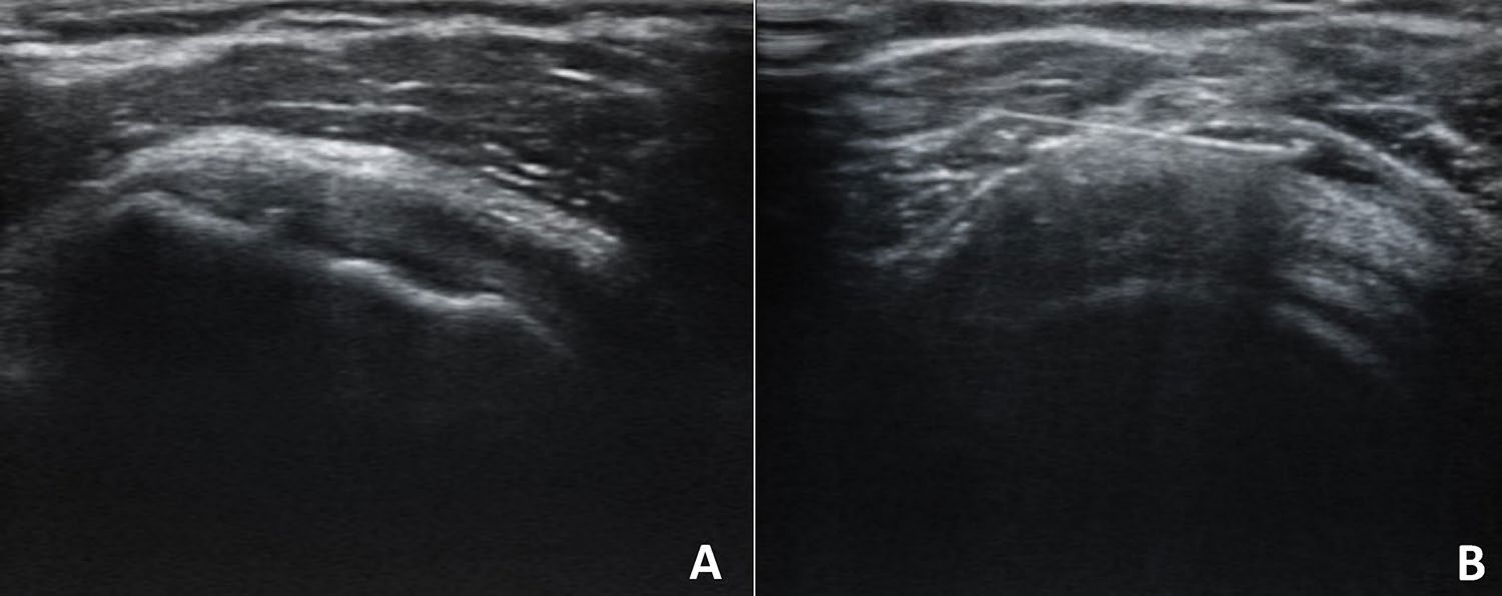
**A** SASD fibroadhesive bursitis; **B** Bursa US-guided hydrodistention

In our case the association between the US-guided capsule distention and the SAD dilatation associated with rehabilitation revealed extremely effective in the treatment of SADS bursitis if associated with adhesive capsulitis [9].
